# Sex-specific *Trans*-regulatory Variation on the *Drosophila melanogaster* X Chromosome

**DOI:** 10.1371/journal.pgen.1005015

**Published:** 2015-02-13

**Authors:** Michael Stocks, Rebecca Dean, Björn Rogell, Urban Friberg

**Affiliations:** 1 Department of Animal and Plant Sciences, University of Sheffield, Sheffield, United Kingdom; 2 Department of Plant Ecology and Evolution, Uppsala University, Uppsala, Sweden; 3 Department of Evolutionary Biology, Uppsala University, Uppsala, Sweden; 4 Department of Genetics, Evolution and Environment, University College London, London, United Kingdom; 5 Department of Animal Ecology, Uppsala University, Uppsala, Sweden; 6 Department of Zoology, Stockholm University, Stockholm, Sweden; 7 IFM Biology, AVIAN Behaviour and Genomics group, Linköping University, Linköping, Sweden; University of Michigan, UNITED STATES

## Abstract

The X chromosome constitutes a unique genomic environment because it is present in one copy in males, but two copies in females. This simple fact has motivated several theoretical predictions with respect to how standing genetic variation on the X chromosome should differ from the autosomes. Unmasked expression of deleterious mutations in males and a lower census size are expected to reduce variation, while allelic variants with sexually antagonistic effects, and potentially those with a sex-specific effect, could accumulate on the X chromosome and contribute to increased genetic variation. In addition, incomplete dosage compensation of the X chromosome could potentially dampen the male-specific effects of random mutations, and promote the accumulation of X-linked alleles with sexually dimorphic phenotypic effects. Here we test both the amount and the type of genetic variation on the X chromosome within a population of *Drosophila melanogaster*, by comparing the proportion of X linked and autosomal *trans*-regulatory SNPs with a sexually concordant and discordant effect on gene expression. We find that the X chromosome is depleted for SNPs with a sexually concordant effect, but hosts comparatively more SNPs with a sexually discordant effect. Interestingly, the contrasting results for SNPs with sexually concordant and discordant effects are driven by SNPs with a larger influence on expression in females than expression in males. Furthermore, the distribution of these SNPs is shifted towards regions where dosage compensation is predicted to be less complete. These results suggest that intrinsic properties of dosage compensation influence either the accumulation of different types of *trans*-factors and/or their propensity to accumulate mutations. Our findings document a potential mechanistic basis for sex-specific genetic variation, and identify the X as a reservoir for sexually dimorphic phenotypic variation. These results have general implications for X chromosome evolution, as well as the genetic basis of sex-specific evolutionary change.

## Author Summary

Theory provides contrasting predictions with respect to the relative magnitude of standing genetic variation on the X chromosome and the autosomes. While most classic population genetics theory suggests a dearth of variation on the X chromosome, theory which concerns selection operating differently in males and females sometimes suggests the opposite. In support of classic theory we find that genetic variants influencing gene expression far from where they are located in the genome (*trans*-acting) are depleted on the X chromosome. *Trans*-acting genetic variants with a larger effect in females than in males do, however, depart from this pattern. Allelic variants with a *trans*-acting female-biased effect are primarily located in regions where the dosage compensation machinery is less active. This suggests that the intrinsic properties of dosage compensation influence the accumulation of different types of *trans*-factors and/or their propensity to accumulate mutations. These findings have general implications for the evolution of the X chromosome, but may also have relevance for the evolution of sexual dimorphism.

## Introduction

When the X chromosome stops recombining with the Y chromosome, a unique genomic environment is formed. Several important population genetic parameters are presumably affected, and collectively this sets the scene for different patterns of evolution on the X chromosome [1–3]. In particular, the level of standing genetic variation on the X chromosome is expected to differ from the autosomes, however, depending upon the precise conditions, either an increase or a decrease is expected.

Classic population genetic theory predicts that the X chromosome will host lower levels of standing genetic variation than the autosomes (e.g. [4–7]). The lower census size of the X chromosome (3/4 of the autosomes) should result in a lower effective population size and thus a higher rate of genetic drift, while hemizygosity in males results in unconditional expression of deleterious mutations. Together, these two features are expected to remove both neutral and deleterious allelic variants at a higher rate on the X chromosome and result in reduced standing genetic variation on the X chromosome relative to the autosomes [6].

Despite these predictions there are nevertheless a range of theoretical arguments why the difference in standing genetic variation between the X and the autosomes could be reduced, or even reversed. In species where neither the X chromosome nor the autosomes recombine in males (e.g. Drosophila), the rate of recombination could be lower on the autosomes. Lower autosomal rates of recombination would, because of Hill-Robertson interference, result in a larger reduction in the effective population size of the autosomes, and consequently a larger reduction in genetic variation [8–11]. Another factor which reduces the effective population size disproportionately on the autosomes is sexual selection on males [12]. Because the X chromosome spends 2/3 of its time in females it is less affected by higher variance in male fitness. In extreme cases sexual selection can theoretically cause the effective population size of the X chromosome to surpass that of the autosomes [12].

Sexually antagonistic selection has also been suggested to increase standing genetic variation on the X chromosome. Theory predicts that hemizygosity of the X chromosome in males, and its presence in females 2/3 of its time, allow for a wider parameter space where allelic variants with sexually opposing effects on fitness can be maintained in polymorphisms [13–15] (but see [16]). The X chromosome has also been suggested to host a disproportionately large fraction of the genome which contributes to sexual dimorphism [14,17–19]. Sexual dimorphism requires elements with sex-specific or sex-limited effects and, because deleterious mutations in such elements are primarily selected in one sex [20], they are predicted to host higher levels of standing genetic variation at mutation-selection-drift balance [21]. However, more recent theory predicts that sexual dimorphism may develop more easily on the autosomes [22].

A further possibility that may affect levels of standing genetic variation on the X chromosome is the way in which dosage compensation (DC) influences the effect size of mutations. Most studies assume an equal effect of mutations on the X and the autosomes [6,7,23,24], but this may be violated when DC is incomplete [6]. Incomplete DC may be more widespread than previously assumed [25]. For example, in mammals, substantial parts of the X chromosome escape DC [26,27] and in several species with ZW sex chromosomes, DC may not occur at all [25,28]. Under incomplete DC, mutations may have smaller phenotypic effects on the X or Z chromosome in the hemizygous sex [6,29]. Deleterious mutations in regions of incomplete DC could therefore experience weaker net purifying selection than mutations in dosage compensated regions. As a consequence, regions of incomplete DC could host more genetic variation.

Genome-wide standing genetic variation has been most extensively studied empirically in *Drosophila melanogaster*. Evidence from this species points to reduced sequence (e.g. [2,30,31]) and transcriptional variation [32] on the X chromosome. In contrast to this, the X chromosome shows no reduction in genetic variation for a range of phenotypic traits [33,34] and seems enriched for sexually antagonistic genetic variation for fitness [35,36]. With respect to sex-bias, genes with female-biased expression are enriched on the X chromosome, while those with male-bias are depleted [3,37,38] (but see [39]), indicating that at least some types of sexual dimorphism develop more easily on the X [1,40]. A number of studies have also compared the distribution of genetic variation in sexual dimorphism over the X and the autosomes for a range of phenotypic traits. The results from these studies are mixed (reviewed in [3,41]). Sex-specific transcriptional variation [42] and general sex-specific genetic variation [43] do however appear enriched on the X chromosome, while the number of eQTLs with a male-specific effect is reduced [44].

In summary there are theoretical as well as empirical reasons to expect depletion as well as enrichment of standing genetic variation on the X chromosome. These predictions depend on the type of mutations and potentially also their location along the X chromosome. To test these predictions we contrast the genomic distribution of *trans*-acting SNPs which associate with sexually concordant and sexually discordant standing genetic variation in gene expression, in a population of *D*. *melanogaster*. We focus on *trans*-regulation because this covers a SNP’s impact on variation within, as well as between, chromosomes. Our results show that SNPs with a sexually concordant effect are significantly depleted on the X chromosome. However, SNPs with a sexually discordant effect are enriched on the X chromosome compared to SNPs with a sexually concordant effect. Furthermore, we show that this relative enrichment of sexually discordant SNPs is driven by SNPs with a female-biased effect and these SNPs tend to accumulate away from regions where DC is initiated and predicted to be strongest. Our results suggest that contrasting patterns of standing genetic variation on the X and autosomes, while influenced by the factors discussed above, might also depend on patterns of DC along the X chromosome.

## Results

To study the chromosomal distribution of SNPs associated in *trans* with sexually concordant and sexually discordant genetic variation in gene expression, we began by reanalyzing the data from Ayroles *et al*. [32]. This dataset consists of gene expression data for males and females from 40 inbred lines of *D*. *melanogaster*, all derived from one large outbred natural population (Raleigh, North America). Using predefined criteria (see methods), we identified one set of genes with expression that shows a high degree of sexually discordant genetic variation (SDV genes, *n* = 121), and another set of genes that shows a high degree of sexually concordant genetic variation (SCV genes, *n* = 151) (S1 Fig.; S1 Table). SDV and SCV genes were evenly distributed, with respect to each other, over the X chromosome and the autosomes (two-proportion z-test: χ^2^ = 0, d.f. = 1, P = 1, S2 Fig.), but they were under-represented on the X chromosome (SCV and SDV genes pooled *vs* all other genes, two-proportion z-test: χ^2^ = 9.5, d.f. = 1, P = 0.002; S2 Fig.).

The inbred lines studied here are all fully sequenced [30]. This allowed us to conduct a genome-wide association study on gene expression independently for each of our selected genes. Since we were exclusively interested in *trans*-regulation (and since *cis-*regulation of these lines has previously been explored [44]), we excluded all identified SNPs located within 10 kb from the 5’ and 3’ ends of the gene in focus (i.e. SNPs associated in cis [44]). This means a *cis*-acting SNP for one gene can still act as a *trans*-acting SNP for other genes. To estimate the X chromosome’s general contribution to *trans*-regulation, and to test for differences between SNPs with sexually concordant and sexually discordant effects, we calculated the proportions of X-linked SNPs associated with SCV and SDV genes, respectively (P-value cut off at 1×10^–5^). Proportions were used to give all genes equal weight. We first conducted tests using all SCV or SDV SNPs, and then divided these into intergenic and genic SNPs. Genic SNPs were further divided into those found in exons and introns. SDV SNPs were in addition divided into those with male- (SDV.M) and female-biased (SDV.F) effect size. Our rationale for this division was that the structural properties of the X chromosome may make it easier for *trans*-regulatory sex-specific factors with a larger effect size on gene expression in either males or females to accumulate. SNPs which control local DC are predicted to have a male-biased effect size, since variation in product from *trans*-regulatory elements, caused by varying degree of DC, should influence variation of the target genes exclusively in males. SNPs located in regions with incomplete DC are, on the other hand, predicted to have a female-biased effect size, because altering both alleles of a *trans*-factor in a homozygous female should have a larger absolute effect on the target gene’s expression than altering the one allele of a hemizygous male.

### *Trans*-acting SNPs with a sexually concordant effect are depleted on the X chromosome

**Fig 1:**
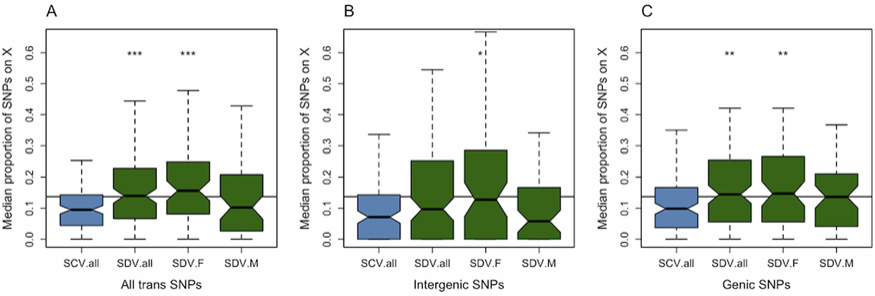
Genomic distribution of *trans*-acting SNPs. Average proportion of SNPs situated on the X chromosome that associate with SCV and SDV genes (A) all *trans*-SNPs (B) intergenic SNPs (C) genic SNPs. SCV.all = all SNPs associated with SCV genes, SDV.all = all SNPs associated with SDV genes, SDV.F = SNPs with female-biased effect size associated with SDV genes, SDV.M = SNPs with male-biased effect size associated with SDV genes. * denotes P < 0.05, ** P < 0.01, *** P < 0.001 comparing SCV.all to SDV SNPs. The line represents the proportion of SNPs on the X chromosome.

The X chromosome contains ~18.8% of the *D*. *melanogaster* genome [30], but only ~13.7% of the SNPs. We use these benchmarks to test if the X chromosome is enriched or depleted of *trans*-regulatory SNPs. We find that SCV SNPs are depleted on the X chromosome with respect to the proportion of SNPs on the X (and thus also with respect to the size of the X) (Table 1, Fig. 1). The proportion of SDV SNPs does not differ from 0.137 (Table 1, Fig. 1). This also applies to the two categories of SDV SNPs (SDV.M and SDV.F), apart from intergenic SDV.M SNPs which are depleted (Table 1, Fig. 1b). While the 95% confidence interval of most classes of SDV.F SNPs clearly overlaps with a proportion of 0.188, this is not the case for any SDV.M SNPs classes (Table 1).

**Table 1:** Proportion of X-linkage for different SNP categories.

SNP class	Gene^1^ type	Median	95% CI^2^	V	P^3^	95% CI^4^	DF	W	P^5^
All trans	SCV	0.095	0.08–0.11	2320	<**0.0001**	-	-	-	-
	SDV.all	0.140	0.13–0.18	4042	0.2111	0.02–0.07	1	9955	**0.0004**
	SDV.F	0.156	0.13–0.21	1539	0.0831	0.03–0.09	1	6083	**0.0003**
	SDV.M	0.102	0.09–0.15	663	0.2723	-0.01–0.05	1	4005	0.4133
- Intergenic	SCV	0.071	0.07–0.10	1787	<**0.0001**	-	-	-	-
	SDV.all	0.097	0.09–0.17	3185	0.5662	-0.00–0.06	1	8273	0.0548
	SDV.F	0.128	0.11–0.22	1212	0.4966	0.0–0.09	1	5020	**0.0122**
	SDV.M	0.058	0.05–0.13	483	**0.0251**	-0.01–0.01	1	3347	0.9286
- Genic	SCV	0.098	0.09–0.12	2786	**0.0002**	-	-	-	-
	SDV.all	0.145	0.13–0.19	4121	0.1012	0.01–0.08	1	9639	**0.0018**
	SDV.F	0.147	0.13–0.20	1490	0.1479	0.01–0.09	1	5752	**0.0057**
	SDV.M	0.137	0.10–0.18	730	0.9177	-0.00–0.06	1	4033	0.1869
- -Exon	SCV	0.053	0.07–0.11	2448	<**0.0001**	-	-	-	-
	SDV.all	0.089	0.11–0.17	3378	0.9098	-0.00–0.05	1	7412	0.0770
	SDV.F	0.105	0.11–0.19	1156	0.7478	-0.00–0.07	1	4137	0.2391
	SDV.M	0.077	0.09–0.18	654	0.7530	-0.00–0.06	1	3346	0.1262
- -Intron	SCV	0.094	0.09–0.12	3021	**0.0013**	-	-	-	-
	SDV.all	0.156	0.13–0.19	4117	0.1031	0.00–0.08	1	9346	**0.0086**
	SDV.F	0.165	0.13–0.21	1551	0.0713	0.00–0.10	1	5599	**0.0169**
	SDV.M	0.123	0.09–0.17	720	0.6778	-0.01–0.06	1	3917	0.3289

P-values for testing whether the median proportion of X-linkage for different SNP categories departs from the expected 0.137, and for testing whether X-linkage differs between SNP categories.

^1^SNPs are divided into those with a sexually concordant (SCV) and sexually discordant (SDV) effect on gene expression. SDV SNPs are further decomposed onto the categories SDV.M or SDV.F, which represents SNPs with a male- or female-biased effect size.

^2^95% CI of the median proportion of X-linked SNPs.

^3^Two-tailed P-values (Wilcoxon test) testing if the X-linked proportion differs from 0.137.

^4^95% CI of the difference in the median proportion of X-linkage for SDV SNP category and SCV SNPs

^5^Two-tailed P-values (Wilcoxon test) testing for a difference in X-linkage between SDV SNP category and SCV SNPs

Note: Not all genes had SNPs that passed the P-value threshold, and some genes had no SNPs which passed the P-value threshold for a certain SNP-class. All *trans* SNPs N = 133 (SCV), N = 119 (SDV); Intergenic SNPs N = 125 (SCV), N = 116 (SDV); Genic SNPs N = 133 (SCV), N = 118 (SDV); Exon SNPs N = 119 (SCV), N = 110 (SDV); Intron SNPs N = 133 (SCV), N = 118 (SDV).

### *Trans*-acting SNPs with a sexually discordant effect are more common on the X-chromosome than SNPs with a sexually concordant effect

When comparing the proportions of SNPs associated with SCV and SDV genes, we find that SDV SNPs are more common on the X chromosome than SCV SNPs (Fig. 1a, Table 1), a result which holds when we relax and strengthen the gene selection criteria (S2 Table). This pattern is primarily driven by genic SNPs (Fig. 1b,c, Table 1) located in introns (Table 1). To test for an enrichment of SDV SNPs with *trans*-acting effects, mediated through *cis*-regulation of X-linked genes, we restricted the intergenic class of SNPs to only include SNPs located 500bp before, or after, a transcription start, or end, site (since most *cis-*regulatory SNPs are found within this region in *D*. *melanogaster* [44]). We found no evidence for such an enrichment (P = 0.76). When we divide the SDV SNPs into SDV.F and SDV.M SNPs, and compare these proportions against the proportion of SCV SNPs, we find that the relative enrichment of SDV SNPs compared to SCV SNPs on the X is mainly driven by SDV.F SNPs (Fig. 1, Table 1). SDV.M SNPs were not significantly more common on the X, but the trend was in the same direction as for female-biased SNPs. The relative effect size of X-linked to autosomal SNPs in general did not differ between SDV and SCV SNPs (S3 Table).

### X-linked SNPs with female-biased effect size are depleted in dosage compensated regions

**Fig 2:**
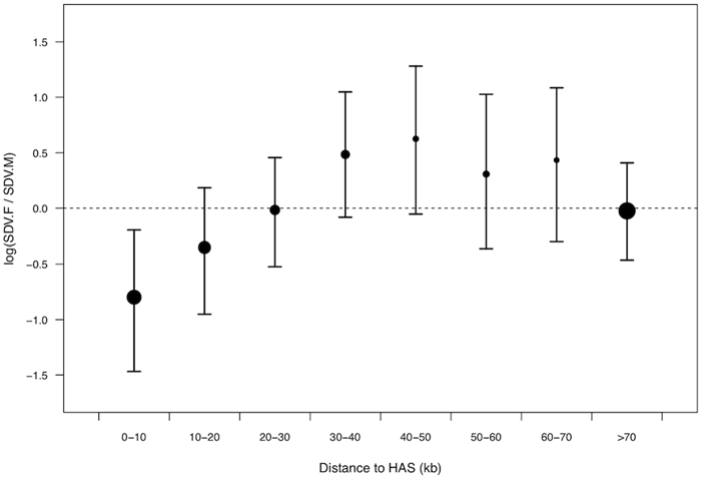
Relative density of female-biased to male-biased X-linked *trans*-acting SNPs in relation to distance to HAS. Dot size corresponds to the average density of male and female-biased SNPs in the specified window and bars represent bootstrapped 95% confidence intervals.

Dosage compensation in *D*. *melanogaster* is initiated at a large number of high affinity sites (HAS) on the X-chromosome, to which the dosage compensation complex (DCC) binds and from where it spreads along the X-chromosome [45]. Dosage compensation of the male X chromosome is therefore expected to be more complete closer to HAS, than further away. Accordingly, we predicted that SDV.F SNPs should be less likely to be found in close proximity to HAS. Using information on the position of HAS from Straub et al [46] we indeed found that SDV.F SNPs lie further from HAS than SDV.M SNPs (Fig. 2, S4 Table). SDV.F SNPs were also located further away from HAS than SCV SNPs (S5 Table). The distribution of SDV.M SNPs, with respect to location of HAS, did not significantly depart from that of SCV SNPs (S5 Table). SDV.F SNPs seem to accumulate at a moderate distance to HAS (Fig. 2).

HAS only function as docking platforms for the DCC, while the means by which the DCC is believed to increase transcription rate at the male X-chromosome is through acetylation of histone H4 at lysine 16 (H4K16ac) [45]. We find complementary evidence that SDV.F SNPs are preferentially located away from regions where DC is predicted to occur when we analyse locations of SNPs with respect to enrichment scores of H4K16ac (S6 Table, S7 Table).

Because DC may not operate in the testis [47] we also removed all gonad specific SDV and SCV genes from the analyses, identified as genes with the highest expression in the testis or the ovaries [48]. Using this subset we still see an over-representation of X-linked SDV SNPs (P = 0.018). When breaking the SDV SNPs down into SDV.F and SDV.M SNPs, we again see an enrichment of SDV.F SNPs on the X (P = 0.0076), but not for SDV.M SNPs (P = 0.86), in line with our previous analyses. In addition, using this subset of genes, SDV.F SNPs are, again, concentrated away from HAS (S3 Fig.).

### Patterns of sex-biased gene expression for X-linked genes with distance to HAS

We next looked at the relationship between the sex-bias of X-linked genes and their distance from HAS, to see if the patterns we find for SDV SNPs are consistent with the sex-bias of X-linked genes. When using whole fly expression data from the DGRP lines we find results consistent with a previous study [49], which showed that X-linked genes become more male-biased with increasing distance to HAS (slope = 1.12 × 10^–6^, P < 0.0001). We also find no significant difference in the sex-bias of genes containing intronic SCV SNPs, SDV SNPs with female-biased (SDV.F) or male-biased (SDV.M) effect size (median sex-biased expression of X-linked gene log_2_(M/F): SDV.M = -0.016, SDV.F = 0.012, SCV = -0.016; Wilcoxon test: SCV-SDV.M P = 0.757, SCV-SDV.F P = 0.083). These results suggest that SDV SNPs with a female-biased effect are not necessarily located in regions with female-biased expression in whole flies. However, Vesenko and Stone [50] recently pointed out that it is problematic to test for an association between sex-biased expression and distance to HAS, with reference to DC, using whole fly expression data. They showed that the positive association disappears when genes with highest expression in the testis, a tissue where DC may not operate [47], are removed. We also confirm this lack of association when testis-biased genes are removed from the whole fly expression data we use (P = 0.069). This result highlights the fact that sex-biased expression is dependent on tissue [51,52]. In our data we have no information on which tissue(s) each of the SDV genes show variation for sex-biased expression in. To limit our analyses to one tissue we repeated the analyses using gene expression in the brain, a shared tissue for which expression data from both males and females is available [53]. Previous analyses of brain [53] and head [54] gene expression data have, in contrary to whole fly gene expression analyses, shown that male-biased genes cluster closer to HAS. In accordance, when we look at the sex-bias of X-linked genes containing SDV and SCV SNPs, we find that genes with intronic SDV.F SNPs are significantly more female-biased than genes with intronic SCV SNPs (median log_2_(M/F): SDV.F = -0.153, SCV = -0.100; Wilcoxon test: P = 0.0117). We find no difference in sex-bias between genes with intronic SDV.M SNPs and genes with intronic SCV SNPs (median log_2_(M/F): SDV.M = -0.113, SCV = -0.100; Wilcoxon test: P = 0.241). Furthermore, we find that X-linked genes containing intronic SDV SNPs become more female-biased with increasing distance to HAS (slope = -1.13 × 10^–6,^ P = 0.009).

### Effect size and distance to HAS

If incomplete DC explains the enrichment of *trans*-acting SNPs with a female-biased effect size on the X (compared to SCV SNPs), we would expect to see a relative decline in SNP effect size in males compared to females with distance to HAS. To test this we separately calculated the regression coefficient between effect size and distance to HAS for each SDV gene, using only significant SDV.M or SDV.F SNPs. In contrast to predictions, we do not find this pattern (median slope: SDV.M SNPs = 1.52 × 10^–7^; SDV.F SNPs = -5.14 × 10^–8^; Wilcoxon test: P = 0.262).

### Effect size and minor allele frequency

Under mutation-selection balance, and the assumption that the deleterious effect of a mutation scales with its effect size, a negative association between effect size and minor allele frequency (MAF) is expected [30,55]. We found the correlation between effect size and MAF to be negative for SCV, SDV.M, as well as SDV.F SNPs (Table 2). The correlation was similar in all three cases, but significantly less negative for SDV.M SNPs.

**Table 2:** Correlation of effect size and minor allele frequency.

Gene type	Median correlation coefficient	95% range	95% CI	W	P
SCV	-0.819	-0.848 to-0.785	-	-	-
SDV.F	-0.830	-0.847 to-0.814	-0.03–0.04	4469	0.7579
SDV.M	-0.770	-0.819 to-0.642	-0.10 - -0.01	2594	**0.0080**

P values represent two-tailed Wilcoxon tests for the difference in correlation coefficients for SCV SNPs and SDV.M or SDV.F SNPs.

### Expression variation in SDV genes is not associated with sequence variation in the sex determination pathway

The predominant view is that all sex-specific gene expression in *D*. *melanogaster* is regulated by sex-specific transcription factors at the terminal end of the sex determination pathway, which interact with *cis*-regulatory elements [56,57]. We therefore investigated whether expression variation in SDV genes is associated with sequence variation in genes within the sex determination pathway. Of the genes in, and associated with, the sex determination pathway (*Sxl*, *tra*, *dsx*, *fru*, *tra-2*, *ix* and *her*) only *Sxl* is located on the X chromosome. Two SDV and two SCV genes had one SNP each in this gene. One SNP is associated with both of the SCV genes and one of the SDV genes. Of the other genes in the sex determination pathway, only *tra*, *dsx* and *fru* had SNPs associated with them. The number of SDV and SCV genes with associated SNPs located in genes in the sex determination pathway was very similar (χ^2^ = 0.6757, d.f. = 1; P = 0.4111). These findings suggest that the sex-specific gene expression variation we observe in our SDV genes is not a result from sequence variation within the sex determination pathway.

### Enrichment of X-linked SDV SNPs is not driven by a few SNPs associated with many genes

To test if the higher proportion of X-linked SDV SNPs with female-biased effect size is driven by a small set of SNPs, each controlling the expression of many SDV genes, or if X-linkage is a more general phenomenon, we investigated whether the number of genes associated with female-biased SNPs was greater than expected by chance. We permuted the number of times a SNP was associated with SDV genes, and calculated the 95% confidence limits of the maximum. We find that three SNPs are associated with more genes than expected by chance (X: 1617959; X: 18634687; X: 18634824 associates with 10, 12, 12 genes respectively; 95% CI of expected maximum distribution: 6–8 genes). These three SNPs are all intergenic and lie between genes CG3795 and Scgdelta (X: 1617959), or CG6873 and CG12609 (X: 18634687, X: 18634824). Removing these three SNPs from our analyses does not change the general conclusion that there is a relative excess of SDV SNPs compared to SCV SNPs on the X chromosome (all SDV SNPs vs SCV SNPs; Wilcoxon test P = 0.0010).

## Discussion

Classic population genetic theory predicts that the X chromosome should be depleted of genetic variation compared to the autosomes, due to a combination of enhanced selection against recessive deleterious mutations and reduced effective population size [4–7]. In accordance with this prediction we find that the X chromosome is depleted of *trans*-acting SNPs with a sexually concordant effect, and that our genes with high transcriptional genetic variation are underrepresented on the X chromosome [32]. However, when we compare SNPs with sexually concordant and sexually discordant *trans*-effects, we observe that the latter are substantially more frequent on the X chromosome and not necessarily depleted. This in particular concerns SNPs with a female-biased effect size, which, depending on site class, are 50–98% more common than SNPs associated with sexually concordant variation. We explore several possible explanations for this pattern.

The relative enrichment of *trans*-acting SNPs with a female-biased effect on the X chromosome compared to SCV SNPs could be interpreted to result from resolved intralocus sexual conflict. Sexual antagonism over expression level is expected to occur for genes on the X and the autosomes alike, but theory shows that the invasion criteria for alleles with a beneficial effect in one sex, and a detrimental effect in the other, are more relaxed on the X chromosome [14] (but see [16]). Accordingly it has been predicted that the X chromosome should be enriched with sexually antagonistic variation [14], which also has been confirmed in *D*. *melanogaster* [35,36,58]. It has also been predicted that enrichment of sexually antagonistic variation would be followed by enrichment of sex-biased genes, as modifiers of gene expression should evolve to reduce expression in the disfavoured sex, which would allow for the sexually antagonistic allele to fix [14].

In line with these predictions, studies have shown that the X chromosome is enriched with female-biased genes in Drosophila [3,37,38]. The same studies do, however, show that enrichment does not apply to male-biased genes. Since theory predicts that sexually antagonistic dominant female beneficial and recessive male beneficial alleles can invade the X chromosome [14], it has been suggested that enrichment of exclusively female-biased genes supports theory, provided that beneficial mutations are in general dominant [49]. Our results can be interpreted in light of this scenario. Dominant female beneficial male detrimental *trans*-acting alleles accumulate on the X chromosome. These *trans*-factors are subsequently followed by modifiers which reduce their impact in males. Mutations in such *trans*-factors will now have a female-biased effect, and because the female-biased *trans*-factors are enriched on the X chromosome, so will SNPs with female-biased effect.

Our finding, that *trans*-SNPs with a female-biased effect size locate away from HAS and away from regions with high intensity of acetylation at H4K16 sites (through which the DCC is believed to achieve increased transcription in males), does however point to a different explanation as to why SNPs with a female-biased effect accumulate on the X chromosome. These results suggest that there are regions of the X chromosome where DC is incomplete, causing female-biased expression, and mutations to have a female-biased effect size. Although our data clearly show that the SNPs with a female-biased effect avoid regions where DC is predicted to be strongest, there are nevertheless a couple of potential caveats with this explanation. In whole fly samples, which we study here, gene expression in general becomes more male-biased, and not female-biased, further away from HAS [49]. This positive association does however disappear when genes with the highest expression in the testis, where DC probably does not occur [47], are removed [50]. This highlights the fact that sex-biased expression, and thus also genetic variation in sex-biased expression, depends on tissue [51,52]. Since our selected genes were identified from whole fly expression data, we have no information on which tissue(s) they show variation in. Looking at the sex-bias of genes in whole fly samples may therefore not accurately reflect what is relevant for our SNPs. When we look at gene expression in a single tissue (brain), which is shared between the sexes and for which there is available data, we do find that intronic female-biased SNPs are more likely associated with female-biased genes than SNPs with a sexually concordant effect. Furthermore the sex-bias of these genes becomes more female-biased with increasing distance to HAS, corroborating a lack of complete DC away from HAS. A second potential problem for the incomplete DC hypothesis is that it relies on the male effect size of SNPs declining with distance to HAS relative to the female effect size. This is a pattern we do not see in our data, but there are at least two explanations which could obscure a decline with distance to HAS. First the effect size of a SNP is measured across all tissues in our whole body samples, and it may therefore not accurately reflect the true effect size in the tissue(s) in which it has its effect, and second, GWAS studies are biased towards finding SNPs with large effect size, preventing SNPs with smaller effect size to influence the association with distance to HAS. With these potential caveats in mind, we next explore what incomplete DC may entail for the evolution of the X chromosome from the perspective of our data.

The most feasible explanation for why we see an enrichment of *trans*-SNPs with female-biased effect size on the X chromosome is that incomplete DC provides genomic regions where expression is naturally female-biased. This results in relatively more X-linked *trans*-factors, and associated mutations, with female-biased effect. Under this scenario the female-bias of *trans*-factors is not adaptive, but simply a consequence of incomplete DC. In addition, incomplete DC is also expected to increase the proportion of X-linked SNPs with female-biased effect size, through reduced intensity of net purifying selection, mediated through a reduced effect size of mutations in males. It has previously been shown that expression of most genes evolves under stabilizing selection [59], and it therefore seems reasonable to assume that this is also the case for *trans*-factors. The negative correlation we observe for all classes of *trans*-SNPs, between effect size and MAF, also supports this view. Assuming that the strength of selection (*s*) against a deleterious mutation is halved in males, when it is located in a region without DC, the equilibrium frequency of a mutation at mutation-selection balance is given by 3*u*/(*s/2*+2*hs*) in a non-dosage compensated region, and 3*u*/(*s*+2*hs*) in a dosage compensated region [10] (where *u* is the mutation rate and *h* is the dominance factor). Since the equilibrium frequency is always higher in regions of incomplete DC, this implies that relatively more SNPs with a female-biased effect size, than those with an equal effect size in both sexes, will segregate at the X chromosome, all else being equal. On the autosomes, the frequency at mutation-selection balance is given by *u*/*sh*, which is always higher than the equilibrium frequency for mutations at dosage compensated regions, and when *h* < 0.5 for mutations in regions without DC. Reduced net purifying selection in regions with incomplete DC thus acts to increase genetic variation on the X chromosome. Given that *h* < 0.5, which is a reasonable assumption since most deleterious mutations are recessive [60,61], the genetic variation in non-dosage compensated regions is not expected to surpass the genetic variation at the autosomes, which is also what we observe. This simplified scenario assumes complete lack of DC, which is probably not the case, but it provides a framework for how to understand how incomplete DC may influence the relative amounts of genetic variation on the autosomes and at different regions of the X chromosome.

Theoretical models concerning standing genetic variation and the rate of evolution of the X chromosome relative to the autosomes primarily focus on dominance [4–7,62] and effective population size [1,63]. However, the possibility that the strength of selection may differ for some classes of mutations between the X chromosome and the autosomes has, to our knowledge, not been empirically considered [64]. Reduced efficacy of selection will, apart from allowing for a higher frequency of deleterious mutations, also result in more frequent fixation of deleterious mutations. Our findings may therefore have relevance for the relative evolutionary rate of the X and the autosomes. Some studies addressing the faster X hypothesis have made efforts to control for gene content (e.g. [65,66]), but also taking incomplete DC into account may be important for a full understanding of the intrinsic differences between mutational effects on the X and the autosomes [1].

An intriguing but speculative possibility is that *trans*-factors have accumulated in regions with incomplete DC, because the natural female-bias in these regions helps them resolve intralocus sexual conflict over gene expression elsewhere in the genome. While the established view suggests that sex, and all somatic sexual differentiation, is exclusively regulated by sex-specific transcription factors or hormones at the terminal end of the sex-determining pathway initiated by one sole master sex-switch gene [56,57], an emerging view suggests that chromosome karyotype also controls sex-differences [54,67–73]. The exact mechanism by which this occurs is not yet understood, but *trans*-factors acting independently from the sex determining pathway, placed in regions with incomplete DC as our findings support, offers an exciting possibility [74].

**Fig 3:**
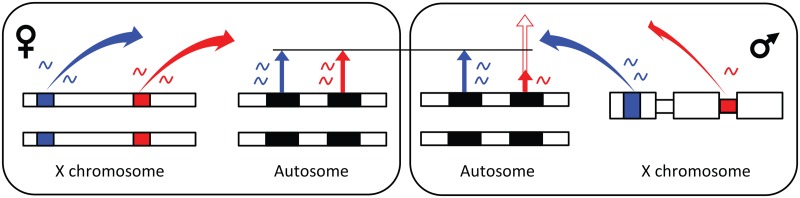
Model for X-linked *trans*-regulation of sex-biased gene expression. Black bars represent genes, coloured bars *trans*-factors, length of arrows from genes indicate amount of gene expression, and colours of arrows match the colour of the *trans*-factor that regulates the expression. *Trans*-factors located in fully dosage compensated regions on the X chromosome (wide parts of male X) are expressed to the same extent in males and females and do not cause sex-biased gene expression of autosomal (and X linked) genes. *Trans*-factors located in regions that fully or partly lack dosage compensation (narrow part of male X) are expressed to a higher extent in females and cause female-biased gene expression when the factor is an activator (solid red gene expression arrow in male), and male-biased gene expression when the factor is a repressor (contour red gene expression arrow in male).

Interestingly, *trans*-factors in regions of incomplete dosage compensation could use the exact same simple mechanism as the master sex-switch gene does in mammals, flies and nematodes, to initiate different signalling cascades in the sexes. These genes are located on one of the sex chromosomes [56,75–77], where they trigger sex differences by a dose effect (presence in one *vs* versus zero copies if it is Y linked and one *vs* versus two copies if it is X linked). This same mechanism would allow *trans*-acting elements to produce female- as well as male-biased gene expression of target genes, as higher expression of an X-linked activator (repressor) of gene expression will cause female- (male-) biased expression (Fig. 3, see also figure 10 in [29]).

Genome-wide gene expression studies comparing expression in males and females of different Drosophila species, show that gene expression changes more rapidly in males compared to females [38,78,79]. This phenomenon is probably also true for many other species, because males are often exposed to sexual selection both when interacting with females and when competing with males, while females are exposed to sexual selection primarily when interacting with males. It thus seems as if males, in general, could benefit more than females from an additional mechanism to control gene expression (males for instance have more cis-regulatory elements than females [44,80]), particularly in species locked into a perpetual arms race between the sexes and male phenotypes. The potential mechanism we describe here instead gives females more opportunity to change than males. However, strong sexual selection on males should often displace the female phenotype from its optimum. The mechanism described here may enable females to maintain their optimum with only minor effects on the male phenotype. In species where males are the homogametic sex (as opposed to the species studied here) there is the opportunity for males to use this mechanism directly, and we would therefore predict a larger Z-linked *trans*-regulatory effect on sex-biased gene expression compared to that observed here. Regulation of sex differences through *trans*-factors located in regions without DC could potentially be part of the explanation as to why we see less DC in ZW sex chromosome systems [81]. If the Z chromosome accumulates *trans*-acting factors which influence gene expression in the direction favoured by males, at the time the W chromosome degrades and loses its gene content, this may reduce selection on females to develop DC in these systems.

The X-linked SNPs we find associated with female-biased transcriptional variation are located in intergenic, and specifically intronic, regions. This suggests that the effect we observe is caused by non-coding *trans*-regulatory factors. The view which has emerged over the last few years suggests that these regions, previously assumed to be inert, are host to a range of non-coding RNAs (ncRNA) with gene regulatory functions [82]. Studies have also shown that introns are particularly enriched with ncRNAs. These studies have foremost been conducted on humans and mice [83–86], but evidence from species of other taxa, such as *Xenopus tropicalis* [87] *Caenorhabditis elegans* [88,89] and *D*. *melanogaster* [88–90], is also accumulating. However, function has only been verified for a small fraction of all non-coding transcripts. Interestingly, we note that two recent studies on mouse have found ncRNAs with female biased expression, which localize to genomic regions which escape X chromosome inactivation [91,92].

Much of what we discuss here relies on the interpretation that *trans*-SNPs with a female-biased effect size are enriched in regions where the DC machinery appears less active, because this should cause *trans*-factors, and thus SNPs within these factors, to have a female-biased effect size. If a *trans*-factor is not female-biased in itself we find it difficult to understand how a mutation can have a female-biased effect. We would nevertheless like to raise the possibility that some idiosyncrasy of DC, that we do not yet understand, is the underlying cause to the intriguing patterns we observe.

In summary we find that *trans*-SNPs with a sexually concordant effect on gene expression are depleted on the X chromosome. We interpret this to primarily result from an exposure of recessive deleterious mutations in males and a reduced effective population size of the X chromosome. The pattern we observe for SCV *trans*-SNPs is in large mirrored by SDV.M SNPs. With respect to SDV.F SNPs we do however find a striking difference, as their genomic distribution is significantly shifted towards that expected on the X chromosome, compared to SCV *trans*-SNPs. This implies that mutations with a female-biased effect size are governed by a population genetics parameter setting different from that of the other types of *trans*-SNPs and/or the presence of other idiosyncrasies of the X chromosome which facilitates their accumulation. Our finding that SDV.F SNPs are enriched in regions where the DC machinery appears less involved opens the possibility for differences between the X and the autosomes that has not been discussed before. Most feasibly the enrichment in regions with predicted incomplete DC is a simple consequence of such regions providing an environment where female-biased expression naturally occurs. In addition, the lower effect size of deleterious mutations in males may reduce the net strength of purifying selection and allow a higher equilibrium frequency of mutations at mutation-selection-drift balance. On a more speculative note, regions with incomplete DC can constitute a platform where *trans*-factors accumulate to directly modify gene expression in a sex-specific manner. This possibility hints that a lack of complete DC should not always be viewed as a problem, but also as an opportunity for the genome to resolve intralocus sexual conflict over gene expression. In closing, we note that the X chromosome in general is depleted of *trans*-acting SNPs with a sexually concordant effect, but that mutations with a *trans*-acting female-biased effect either occur more frequently on the X chromosome or have the capacity to counterbalance the factors which mediate erosion of genetic variation on the X chromosome.

## Methods

### Data

We used whole body microarray data from 40 inbred lines of *D*. *melanogaster* (the DGRP lines from the Raleigh population) from the study by Ayroles et al [32]. The raw data were downloaded from http://www.ebi.ac.uk/arrayexpress/experiments/E-MEXP-1594 and normalized using RMA [93]. SNP data for these lines was taken from Mackay et al [30].

### Gene selection criteria

We identified two classes of genes: those with genetic variation for sexually discordant (SDV genes) and those with sexually concordant genetic variation (SCV genes). To accomplish this we first fit a linear mixed model using Restricted Maximum Likelihood (REML) of gene expression levels independently for all genes, specifying Sex (fixed factor), Line (random factor) and Sex × Line (random factor) as predictors. From these models we extracted the Line and the Line × Sex variance components to create a variation index (I), that measures the percentage contribution of the Sex × Line variance to the total genetic variance [I = V_Sex × Line_ /(V_Line_ + V_Sex × Line_)]. We then classified genes as SDV or SCV according to the following characteristics: Genes with an I > 0.95 and a large (> 0.2) and significant (P < 0.0001) Sex × Line variance component were classified as SDV, and genes with an I < 0.05 and a large (> 0.2) and significant (P < 0.0001) Line variance component were classified as SCV. Using this procedure, 121 genes were classified as SDV and 152 genes as SCV (S1 Fig.; S1 Table). To verify that our procedure captured genes with sexually discordant and concordant variation we calculated the intersexual genetic correlation *r*_MF_ for SDV and SCV genes. SDV genes, on average (± standard deviation), had a low *r*_MF_ (-0.03 ± 0.15), whereas SCV genes had a high average *r*_MF_ (0.93 ± 0.07). 98% (mean abs[log_2_{male/female}] expression ± SD is 0.63 ± 0.39) of the SDV genes and 49% (0.07 ± 0.06) of the SCV genes had sex-biased expression at a P-value < 0.05.

### GWAS for gene expression of selected genes

For each SDV and SCV gene, mean gene expression for each Line was calculated independently for each sex. Gene expression values (G.E) for each gene were uploaded onto the DGRP website (http://dgrp.gnets.ncsu.edu/) to identify SNPs. Analyses include only those SNPs with two alleles and where the minor allele appears in at least 10 percent of the lines. A linear mixed model for each SNP was run using the model G.E = mean + S + M × S + L(M) + E where M is the Marker (SNP), S is Sex, L(M) is the random Line effect nested within Marker and E is Error.

### SNP selection criteria

To contrast SNPs with a sex-specific effect on expression to SNPs with a concordant effect we only retained SNPs with concordant association with gene expression across the sexes for SCV genes (i.e. significant pooled P-value across the sexes) and only SNPs with a sex-specific association with gene expression for SDV genes (i.e. significant SNP × Sex P-value). Since we were interested in the chromosomal distribution of SNPs rather than identifying particular SNPs with association beyond doubt, there is sequence and gene expression data for only 40 of the DRGP lines, and since *trans*-SNPs have been shown to have relatively small effect size and escape detection when using too stringent criteria [94], we chose a P-value cut off at 1×10^–5^. This P-value cut off corresponds to a median FDR of 0.19 per SCV gene and 0.27 per SDV gene. This slightly higher FDR for SNPs associated with FDR genes rendered our results conservative, if anything, since the estimated true proportions of SDV and SCV SNPs (0.085 and 0.141) that associate with the X chromosome differ more than the observed proportions (0.095 and 0.140). We calculated the expected true proportions using the formula [observed proportion X-linked SNPs] = FDR x [proportion X-linked SNPs of all tested SNPs] + [1-FDR] x [true proportion of X-linked SNPs], where [proportion X-linked SNPs of all tested SNPs] = 0.137.

To control for differences in linkage between the autosomes and the X chromosome we grouped together, and counted as one SNP, any SNPs that associated with a particular gene that were within 10 base pairs of each other on the autosomes and 30 base pairs of each other on the X chromosome. The different distances standardised the linkage between the autosomes and X chromosome to an *r* ^2^ of 0.2 [30].

### Genomic distribution and effect size of SNPs associating with SDV and SCV genes

For each gene we calculated the proportion of SNPs on the X chromosome and the relative effect size of SNPs on the X chromosome (average effect size of SNPs on X / average effect size across all chromosomes). SDV SNPs were classified as either female- or male-biased based on the sex the SNP had the larger effect size in. The proportion of male- and female-biased SNPs associating with SDV genes was compared with all SNPs associating with SCV genes (their sex-bias was approximately 0, given that these genes were chosen to have a very low sex-specific genetic variation and that only SNPs with a concordant effect on gene expression in the two sexes was chosen). All analyses were conducted in R v2.13.0 [95].

## Supporting Information

S1 Fig Visual representation of studied genes according to their sex-specific genetic variance (y-axis) and sexually concordant genetic variance (x-axis) in gene expression. Genes classified as having primarily sexually discordant variation (SDV genes, n = 121) are represented in green and genes classified as having primarily sexually concordant variation (SCV genes, n = 151) are represented in blue. Remaining genes are represented in grey. The one gene that departs from the general pattern in the figure did not fulfil significant Line x Sex variation. Axes are log10(x+1) transformed. See methods for the selection criteria that were used.
(DOCX)

S2 Fig Genomic distribution of SDV and SCV genes with respect to the X chromosome and the autosomes (chromosomes 2, 3 and 4). (DOCX)

S3 Fig Relative density of female-biased to male-biased X-linked *trans*-acting SNPs in relation to distance to HAS, excluding genes with maximum expression in the gonad. Dot size corresponds to the average density of male and female-biased SNPs in the specified window and bars represent bootstrapped 95% confidence intervals.
(DOCX)

S1 Table List of SDV and SCV genes with Flybase gene identity. (CSV)

S2 Table Average proportion of SNPs on the X chromosome that associate with SCV and SDV genes, under different gene selection criteria. See Methods for a detailed description of selection criteria.
(DOCX)

S3 Table Average effect size of SNPs on the X chromosome compared to autosomal SNPs. (DOCX)

S4 Table Median distance to HAS of male-biased (SDV.M) and female-biased (SDV.F) SNPs that associate with SDV genes. (DOCX)

S5 Table Distance to HAS of SNPs that associate with SCV and SDV genes. For SDV genes, SNPs are classified as either male- (SDV.M) or female-biased (SDV.F) based on male and female effect sizes.
(DOCX)

S6 Table Median SNP acetylation enrichment of male-biased (SDV.M) and female-biased (SDV.F) SNPs that associate with SDV genes. (DOCX)

S7 Table Median acetylation enrichment of SNPs that associate with SCV and SDV genes. For SDV genes, SNPs are classified as either male- (SDV.M) or female-biased (SDV.F) based on male and female effect sizes.
(DOCX)
