# Supplementary material for: Sex-specific Trans-regulatory Variation on the Drosophila melanogaster X Chromosome
Source: PLoS Genet. 2015 Feb 13;11(2):e1005015. doi: 10.1371/journal.pgen.1005015 (PMC4334168; doi:10.1371/journal.pgen.1005015)
Supplement: S2 Table — See Methods for a detailed description of selection criteria. (DOCX) [file pgen.1005015.s005.docx]

|  | | Sex*Line > 0.2 (SDV),  L > 0.2 (SCV) | | Sex*Line > 0.25 (SDV), L > 0.25 (SCV) | |
| --- | --- | --- | --- | --- | --- |
|  |  | Median | P value | Median | P value |
| I > 0.90 (SDV)  I < 0.10 (SCV) | SCV | 0.095 | a | 0.095 | d |
|  | SDV-all | 0.130 | **0.0005** | 0.132 | **0.0004** |
|  | SDV-F | 0.157 | **0.0001** | 0.159 | **0.0001** |
|  | SDV-M | 0.107 | 0.4343 | 0.109 | 0.3018 |
| I > 0.95 (SDV)  I < 0.05 (SCV) | SCV | 0.095 | b | 0.095 | e |
|  | SDV-all | 0.140 | **0.0004** | 0.143 | **0.0003** |
|  | SDV-F | 0.156 | **0.0003** | 0.157 | **0.0004** |
|  | SDV-M | 0.102 | 0.4133 | 0.124 | 0.2038 |
| I > 0.98 (SDV)  I < 0.25 (SCV) | SCV | 0.103 | c | 0.101 | f |
|  | SDV-all | 0.143 | **0.0421** | 0.154 | **0.0423** |
|  | SDV-F | 0.157 | **0.0186** | 0.157 | **0.0304** |
|  | SDV-M | 0.115 | 0.9801 | 0.126 | 0.6507 |

Note: Only data for all trans SNPs presented. Number of genes with SNPs associated

a SDV = 148, SCV = 140; b SDV = 119, SCV = 133; c SDV = 108, SCV = 48; d SDV = 117, SCV = 126; e SDV = 97, SCV = 122; f SDV = 87, SCV = 40
