# Supplementary material for: Sex-specific Trans-regulatory Variation on the Drosophila melanogaster X Chromosome
Source: PLoS Genet. 2015 Feb 13;11(2):e1005015. doi: 10.1371/journal.pgen.1005015 (PMC4334168; doi:10.1371/journal.pgen.1005015)
Supplement: S3 Table — (DOCX) [file pgen.1005015.s006.docx]

| **SNP class** | **Gene type** | **Median** | **95% CI** | **DF** | **Wilcoxon** | **P** |
| --- | --- | --- | --- | --- | --- | --- |
| All trans | SCV | 0.997 | - | - | - | - |
|  | SDV-all | 0.999 | -0.01-0.02 | 1 | 6328 | 0.4580 |
|  | SDV-F | 0.994 | -0.02-0.13 | 1 | 3268 | 0.6934 |
|  | SDV-M | 1.000 | -0.00-0.03 | 1 | 3064 | 0.1173 |
| - Intergenic | SCV | 1.000 | - | - | - | - |
|  | SDV-all | 0.994 | -0.03-0.01 | 1 | 3107 | 0.3371 |
|  | SDV-F | 0.993 | -0.04-0.01 | 1 | 1654 | 0.2445 |
|  | SDV-M | 0.995 | -0.03-0.02 | 1 | 1448 | 0.7161 |
| - Genic | SCV | 0.991 | - | - | - | - |
|  | SDV-all | 0.999 | -0.01-0.02 | 1 | 5893 | 0.311 |
|  | SDV-F | 0.989 | -0.02-0.01 | 1 | 2887 | 0.5713 |
|  | SDV-M | 1.005 | 0.00-0.04 | 1 | 3059 | **0.0161** |
| - - Exon | SCV | 0.998 | - | - | - | - |
|  | SDV-all | 1.005 | -0.02-0.03 | 1 | 2968 | 0.4577 |
|  | SDV-F | 1.000 | -0.02-0.04 | 1 | 1521 | 0.5612 |
|  | SDV-M | 1.005 | -0.02-0.04 | 1 | 1447 | 0.5343 |
| - - Intron | SCV | 0.989 | - | - | - | - |
|  | SDV-all | 0.997 | -0.01-0.02 | 1 | 4842 | 0.5937 |
|  | SDV-F | 0.992 | -0.03-0.01 | 1 | 2407 | 0.5147 |
|  | SDV-M | 1.000 | -0.00-0.03 | 1 | 2458 | 0.1256 |

**Note:** Not all genes had SNPs that passed the P-value threshold. All *trans* SNPs N = 133 (SCV), N = 119 (SDV); Intergenic SNPs N = 125 (SCV), N = 116 (SDV); Genic SNPs N = 133 (SCV), N = 118 (SDV); Exon SNPs N = 119 (SCV), N = 110 (SDV); Intron SNPs N = 133 (SCV), N = 118 (SDV). P values (two-sided) denote Wilcoxon test comparing SCV to SDV-all, SCV to SDV-M or SCV to SDV-F.
