# Supplementary material for: Sex-specific Trans-regulatory Variation on the Drosophila melanogaster X Chromosome
Source: PLoS Genet. 2015 Feb 13;11(2):e1005015. doi: 10.1371/journal.pgen.1005015 (PMC4334168; doi:10.1371/journal.pgen.1005015)
Supplement: S4 Table — (DOCX) [file pgen.1005015.s007.docx]

| **SNP class** | **SNP type** | **10^th^ percentile distance** | **P value 10^th^ percentile** | **25^th^ percentile distance** | **P value 25^th^ percentile** | **Median distance** | **P value median** |
| --- | --- | --- | --- | --- | --- | --- | --- |
| All trans | SDV-M | 9458 | - | 15366 | - | 31620 | - |
|  | SDV-F | 25910 | **<0.0001** | 29384 | **0.0013** | 41649 | **0.0279** |
| - Intergenic | SDV-M | 19039 | - | 26227 | - | 45195 | - |
|  | SDV-F | 29541 | **0.0454** | 33056 | 0.142 | 41080 | 0.9659 |
| - Genic | SDV-M | 10022 | - | 16185 | - | 28527 | - |
|  | SDV-F | 20816 | **0.0003** | 27399 | **0.0032** | 36360 | 0.1169 |
| - - Exon | SDV-M | 12422 | - | 15069 | - | 17552 | - |
|  | SDV-F | 21107 | 0.0751 | 21107 | 0.1311 | 25432 | 0.2794 |
| - - Intronic | SDV-M | 13854 | - | 21681 | - | 34629 | - |
|  | SDV-F | 29263 | **<0.0001** | 40238 | **0.0005** | 52439 | 0.0603 |

Note: Median values are presented for 10^th^ percentile, 25^th^ percentile and median distance per gene. P values (two-sided) denote Wilcoxon test comparing SDV-M to SDV-F.
