# Supplementary material for: Sex-specific Trans-regulatory Variation on the Drosophila melanogaster X Chromosome
Source: PLoS Genet. 2015 Feb 13;11(2):e1005015. doi: 10.1371/journal.pgen.1005015 (PMC4334168; doi:10.1371/journal.pgen.1005015)
Supplement: S5 Table — For SDV genes, SNPs are classified as either male- (SDV.M) or female-biased (SDV.F) based on male and female effect sizes. (DOCX) [file pgen.1005015.s008.docx]

| **SNP class** | **SNP type** | **10^th^ percentile distance** | **P value 10^th^ percentile** | **25^th^ percentile distance** | **P value 25^th^ percentile** | **Median distance** | **P value median** |
| --- | --- | --- | --- | --- | --- | --- | --- |
| All trans | SCV | 11562 | - | 19995 | - | 32763 | - |
|  | SDV-M | 9458 | 0.1502 | 15366 | 0.4419 | 31620 | 0.4713 |
|  | SDV-F | 25910 | **0.0002** | 29384 | **0.0024** | 41649 | 0.0666 |
| - Intergenic | SCV | 17211 | - | 26920 | - | 39862 | - |
|  | SDV-M | 19039 | 0.4480 | 26227 | 0.9247 | 45195 | 0.6864 |
|  | SDV-F | 29541 | **0.0007** | 33056 | **0.0257** | 41080 | 0.6637 |
| - Genic | SCV | 11562 | - | 17948 | - | 31219 | - |
|  | SDV-M | 10022 | 0.2626 | 16185 | 0.5096 | 28527 | 0.6449 |
|  | SDV-F | 20816 | **0.0010** | 27399 | **0.0084** | 36360 | 0.1824 |
| - - Exon | SCV | 5117 | - | 7091 | - | 13252 | - |
|  | SDV-M | 12422 | 0.1013 | 15069 | 0.0736 | 17552 | 0.1352 |
|  | SDV-F | 21107 | **0.0010** | 21107 | **0.0018** | 25432 | **0.0151** |
| - - Intronic | SCV | 16491 | - | 23842 | - | 35742 | - |
|  | SDV-M | 13854 | 0.0605 | 21681 | 0.1441 | 34629 | 0.4926 |
|  | SDV-F | 29263 | **0.0018** | 40238 | **0.0077** | 52439 | 0.1378 |

Note: Median values are presented for 10^th^ percentile, 25^th^ percentile and median distance per gene. P values (two-sided) denote Wilcoxon test comparing SCV to SDV-M or SDV-F.
