# Supplementary material for: Sex-specific Trans-regulatory Variation on the Drosophila melanogaster X Chromosome
Source: PLoS Genet. 2015 Feb 13;11(2):e1005015. doi: 10.1371/journal.pgen.1005015 (PMC4334168; doi:10.1371/journal.pgen.1005015)
Supplement: S6 Table — (DOCX) [file pgen.1005015.s009.docx]

| **SNP class** | **SNP type** | **90^th^ percentile** | **P value 90^th^ percentile** | **75^th^ percentile** | **P value 75^th^ percentile** | **Median** | **P value median** |
| --- | --- | --- | --- | --- | --- | --- | --- |
| All trans | SDV-M | 11.00 | - | 9.66 | - | 6.20 | - |
|  | SDV-F | 9.14 | **0.0046** | 7.92 | **0.0309** | 5.56 | 0.3484 |
| - Intergenic | SDV-M | 7.89 | - | 7.25 | - | 5.60 | - |
|  | SDV-F | 6.83 | 0.2109 | 6.19 | 0.2851 | 4.79 | 0.5094 |
| - Genic | SDV-M | 11.74 | - | 10.23 | - | 6.40 | - |
|  | SDV-F | 9.25 | **0.0047** | 7.71 | **0.0319** | 6.36 | 0.6302 |
| - - Exon | SDV-M | 12.59 | - | 12.22 | - | 10.88 | - |
|  | SDV-F | 10.68 | **0.0049** | 9.90 | **0.0065** | 9.12 | **0.0379** |
| - - Intronic | SDV-M | 9.16 | - | 7.27 | - | 5.16 | - |
|  | SDV-F | 7.08 | **0.0119** | 6.12 | 0.0911 | 4.52 | 0.2898 |

Note: Median values are presented for 90^th^ percentile, 75^th^ percentile and median distance per gene. P values (two-sided) denote Wilcoxon test comparing SDV-M to SDV-F.
