# Supplementary material for: Sex-specific Trans-regulatory Variation on the Drosophila melanogaster X Chromosome
Source: PLoS Genet. 2015 Feb 13;11(2):e1005015. doi: 10.1371/journal.pgen.1005015 (PMC4334168; doi:10.1371/journal.pgen.1005015)
Supplement: S7 Table — For SDV genes, SNPs are classified as either male- (SDV.M) or female-biased (SDV.F) based on male and female effect sizes. (DOCX) [file pgen.1005015.s010.docx]

| **SNP class** | **SNP type** | **90^th^ percentile** | **P value 90^th^ percentile** | **75^th^ percentile** | **P value 75^th^ percentile** | **Median** | **P value median** |
| --- | --- | --- | --- | --- | --- | --- | --- |
| All trans | SCV | 10.4 | - | 8.37 | - | 5.91 | - |
|  | SDV-M | 11.00 | 0.2836 | 9.66 | 0.1183 | 6.20 | 0.2410 |
|  | SDV-F | 9.14 | 0.0538 | 7.92 | 0.2994 | 5.56 | 0.9262 |
| - Intergenic | SCV | 8.25 | - | 7.02 | - | 4.49 | - |
|  | SDV-M | 7.89 | 0.4562 | 7.25 | 0.9535 | 5.60 | 0.3131 |
|  | SDV-F | 6.83 | **0.0119** | 6.19 | 0.1428 | 4.79 | 0.5852 |
| - Genic | SCV | 11.09 | - | 8.72 | - | 5.91 | - |
|  | SDV-M | 11.74 | 0.1627 | 10.23 | 0.1015 | 6.40 | 0.7117 |
|  | SDV-F | 9.25 | 0.0698 | 7.71 | 0.3158 | 6.36 | 0.9387 |
| - - Exon | SCV | 12.72 | - | 11.89 | - | 9.96 | - |
|  | SDV-M | 12.59 | 0.9712 | 12.22 | 0.5416 | 10.88 | 0.1750 |
|  | SDV-F | 10.68 | **0.0030** | 9.90 | **0.0112** | 9.12 | 0.4364 |
| - - Intronic | SCV | 8.10 | - | 6.89 | - | 4.20 | - |
|  | SDV-M | 9.16 | 0.3287 | 7.27 | 0.5899 | 5.16 | 0.4129 |
|  | SDV-F | 7.08 | 0.0602 | 6.12 | 0.1049 | 4.52 | 0.9654 |

Note: Values presented are the median of the 90^th^ percentile, 75^th^ percentile and median distance per gene. P values (two-sided) denote Wilcoxon test comparing SCV to SDV-M or SDV-F.
