# Supplementary material for: Sex-specific Trans-regulatory Variation on the Drosophila melanogaster X Chromosome
Source: PLoS Genet. 2015 Feb 13;11(2):e1005015. doi: 10.1371/journal.pgen.1005015 (PMC4334168; doi:10.1371/journal.pgen.1005015)
Supplement: Supplementary file 8 [file pgen.1005015.s001.docx]

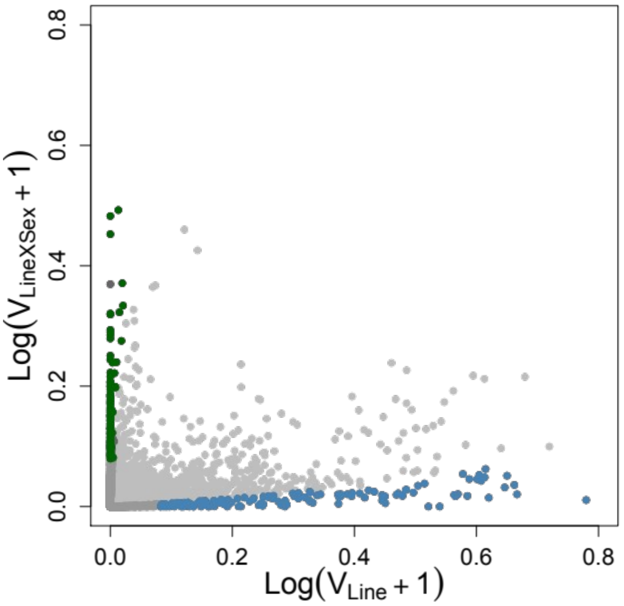

0.0
0.2
0.4
0.6
0.8
$\text{Log}(V_{\text{Line}} + 1)$
$\text{Log}(V_{\text{LineXSex}} + 1)$
