# Supplementary material for: Sex-specific Trans-regulatory Variation on the Drosophila melanogaster X Chromosome
Source: PLoS Genet. 2015 Feb 13;11(2):e1005015. doi: 10.1371/journal.pgen.1005015 (PMC4334168; doi:10.1371/journal.pgen.1005015)
Supplement: Supplementary file 9 [file pgen.1005015.s002.docx]

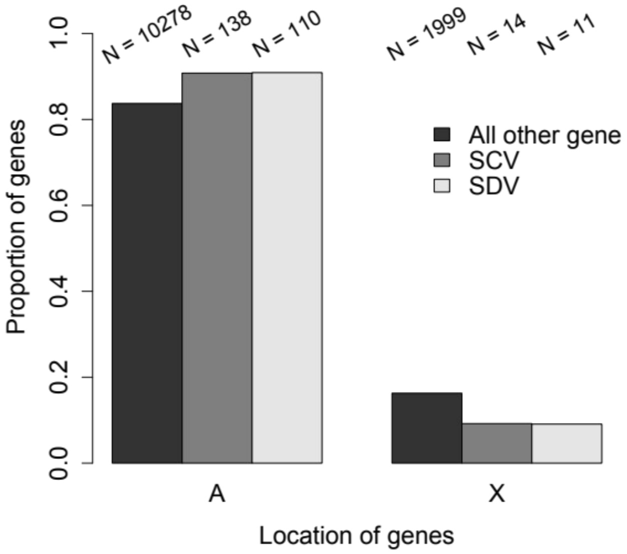

N = 10278
N = 138
N = 110
N = 1999
N = 14
N = 11
1.0
0.8
0.6
0.4
0.2
0.0
Proportion of genes
All other gene
SCV
SDV
A
X
Location of genes
