# Supplementary material for: Sex-specific Trans-regulatory Variation on the Drosophila melanogaster X Chromosome
Source: PLoS Genet. 2015 Feb 13;11(2):e1005015. doi: 10.1371/journal.pgen.1005015 (PMC4334168; doi:10.1371/journal.pgen.1005015)
Supplement: Supplementary file 10 [file pgen.1005015.s003.docx]

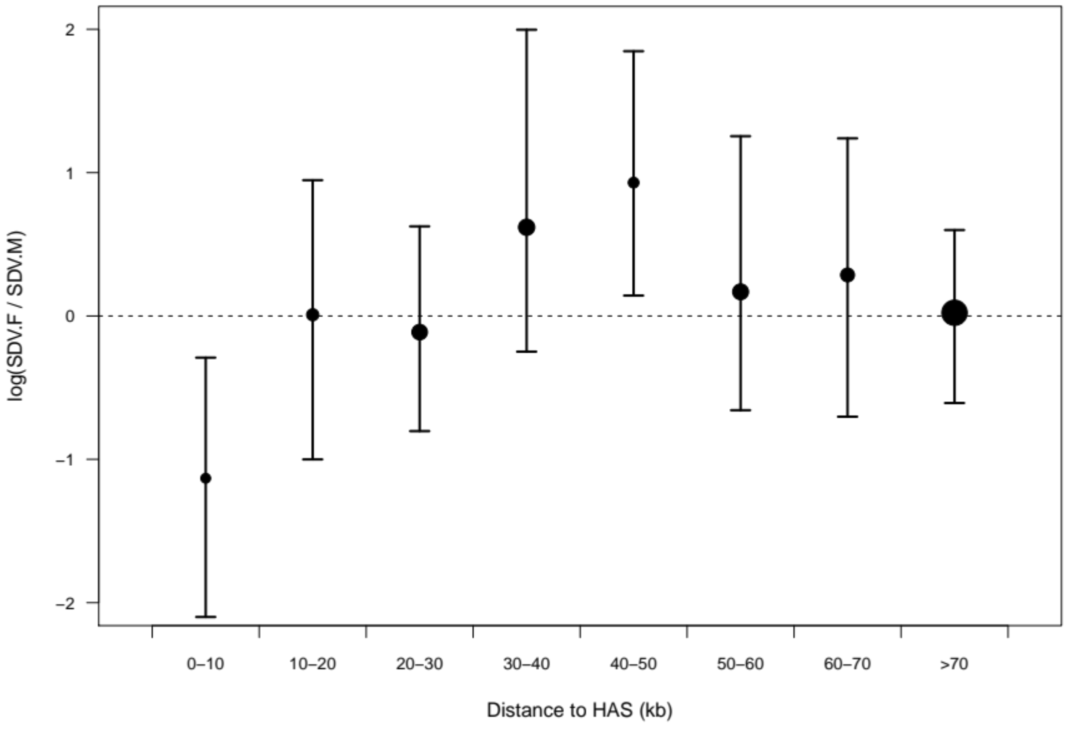
log(SDV.F / SDV.M)
2
1
0
−1
−2
0–10
10–20
20–30
30–40
40–50
50–60
60–70
>70
Distance to HAS (kb)
